# On the background of plastics nanoparticles in our research

**DOI:** 10.3389/ftox.2026.1653106

**Published:** 2026-04-23

**Authors:** Martin Lundqvist, Jing Hua, Mikael T. Ekvall, Tommy Cedervall

**Affiliations:** Biochemistry and Structural Biology, Lund University, Lund, Sweden

**Keywords:** plastic background, nanoplastics, nanoparticles, particle release, ultrasound, test tubes, sonication

## Abstract

We must recognize that plastics likely constitute a pervasive background in many environments. Beyond their previously documented risks to ecosystems and human health, plastics may also exert unintended influences on laboratory experiments, potentially distorting experimental outcomes. Here, we demonstrate that ultrasound treatment of commonly used laboratory test tubes releases nanoplastics into ultrapure water, reaching concentrations of up to 1–2 × 10^10^ particles mL^-1^ during water bath sonication, and we hypothesize how such contamination may affect experimental results.

## Introduction

The environmental impact of nano- and microplastics has become an increasing concern over the past decade, with numerous studies highlighting their potential ecotoxicity (Du et al., 2021; Ge et al., 2021; Yang et al., 2025). Concurrently, mounting evidence indicates that plastic micro- and nanoparticles have permeated nearly every compartment of modern life. They have been detected in food and beverages, such as tea (Hernandez et al., 2019), and bottled water (Vega-Herrera et al., 2023); in environmental matrices including oceans (ten Hietbrink et al., 2025), and air (Chen et al., 2020); and even in human organs and blood (Jenner et al., 2022; Ragusa et al., 2021; Leonard et al., 2024). Given this pervasive presence of plastics across macro-, micro-, and nano-scales, it is increasingly evident that plastics also constitute an unavoidable background in laboratory environments.

This raises the concern that many experiments conducted over past decades may have been performed in the presence of an unrecognized plastic background. Importantly, this background may not be limited to the macroscopic plastic of laboratory vessels, but may also include colloidally stable, irregularly shaped nanoparticles released from plastic surfaces. Such particles could introduce unintended experimental artifacts, as many chemical and biological processes are surface-sensitive and can be strongly influenced by the presence of nanoscale interfaces (Bhan and Delgass, 2022; Chandra et al., 2020; Dusselier and Sels, 2014; Li et al., 2018; Vogt and Weckhuysen, 2022; Zhu et al., 2017). Indeed, interactions between proteins and plastic surfaces are well documented (Lynch et al., 2007).

**FIGURE 1:**
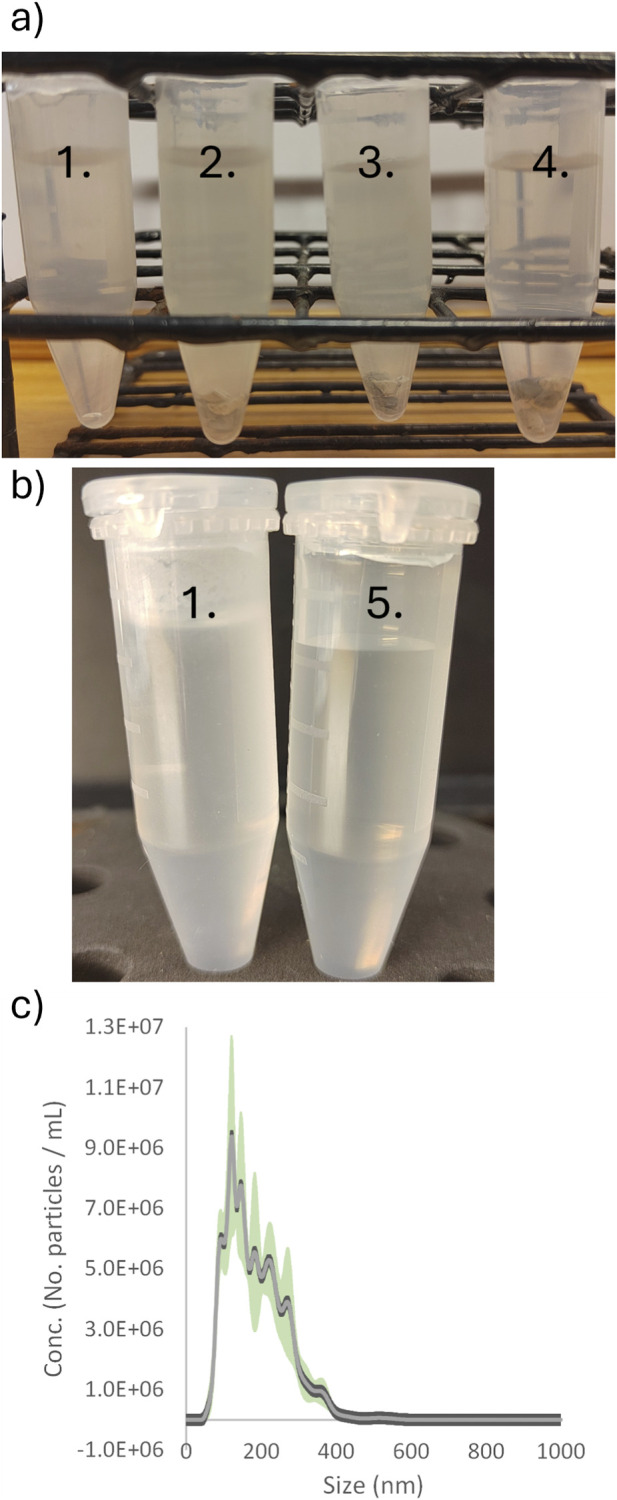
Pictures and Nanoparticle tracking analysis (NTA) data for ultrasound treated 5 mL test tube containing MilliQ-H_2_O. **(a)** Control sample of MilliQ-H_2_O treated with ultrasound (marked 1, left). Three additional samples of MilliQ-H2O containing EPDM granules were subjected to different treatments: ultrasound (marked 2), stirring with a magnetic bar (marked 3), and rocking (marked 4). The control sample (1) appears cloudier than the rocked sample with granules (4). **(b)** Ultrasound-treated control sample (marked 1) compared to an untreated control (marked 5). **(c)** NTA data from sample 1. The light gray line shows the mean particle size distribution from five individually recorded videos, and the green shading indicates the standard deviation.

Evidence for this phenomenon emerged during the setup of experiments designed to study the release of small molecules and particles from ethylene propylene diene monomer (EPDM) granules subjected to mechanical agitation (rocking, magnetic stirring, and ultrasound). As a control, a 5 mL plastic tube containing just ultra-pure (Milli-Q) water was subjected to the same ultrasound treatment (Figures 1a,b). Unexpectedly, the control sample exhibited greater turbidity after sonication than samples containing EPDM granules subjected to rocking.

We demonstrate that commonly used plastic laboratory test tubes can release plastic nanoparticles—here referred to as nanoplastics, defined as non-engineered plastic particles smaller than 1 µm (Gigault et al., 2021; Gigault et al., 2018), during routine experimental procedures.

## Materials and methods

### Tubes tested

The plastic tubes, made of polypropylene see Supplementary Figure S1 and Supplementary Table S1, were purchased from Sarstedt, 1.5, 2, 15 and 50 mL (Sarstedt.com) and Eppendorf, 5 mL (Eppendorf.com).

### Ultrasound treatment

The tubes were filled with 4 mL of MilliQ H_2_O, submerged in an ultrasound bath, and held in place so that the lid was approximately 1 cm above the water surface. The first treatment was conducted using an ultrasound bath from Struers (Copenhagen, Denmark), which was old and lacking in detailed operating specifications. The total treatment time was 30 h. Figure 1 shows the tubes after the treatment alongside a control sample that was not exposed to ultrasound.

The second treatment was carried out using an Elmasonic P 60 H ultrasound bath, set to 100% power and a frequency of 37 kHz. Tubes containing MilliQ H_2_O were subjected to 3 h of ultrasound treatment per day for 5 consecutive days, followed by a 2-day resting period. This cycle was repeated three times, resulting in a total of 45 h of ultrasound treatment.

### Particle characterization

#### Nanoparticle tracking analysis (NTA)

NTA was performed using a NanoSight LM10 instrument equipped with a 405 nm laser. For each sample, five videos were recorded with the camera level set to 14. The videos were analyzed using NTA software version 3.1.

#### Fourier transform InfraRed spectroscopy (FTIR)

FTIR analysis was performed using a Nicolet iS5 spectrometer equipped with an iD5 ATR unit (Thermo Scientific). A volume of 6 µL of the sample was pipetted onto the crystal in the ATR unit and allowed to dry for 30 min before recording the spectrum. Spectra were collected in the range of 4,000–550 cm^-1^ using 64 scans at a resolution of 4 cm^-1^.

Spectral analysis was carried out using PerkinElmer Spectrum IR software (version 10.7.2.1630), and the spectra were compared against a polymer library, polyatr, containing 159 reference IR spectra.

## Results

The control sample was analyzed with NTA, see Figure 1 panel c. The result shows that the MilliQ-H_2_O in the control sample contains a significant number of nanoparticles after the ultrasound treatment. Hence, the sample tube has released nanoparticles into the MilliQ-H_2_O.

**FIGURE 2:**
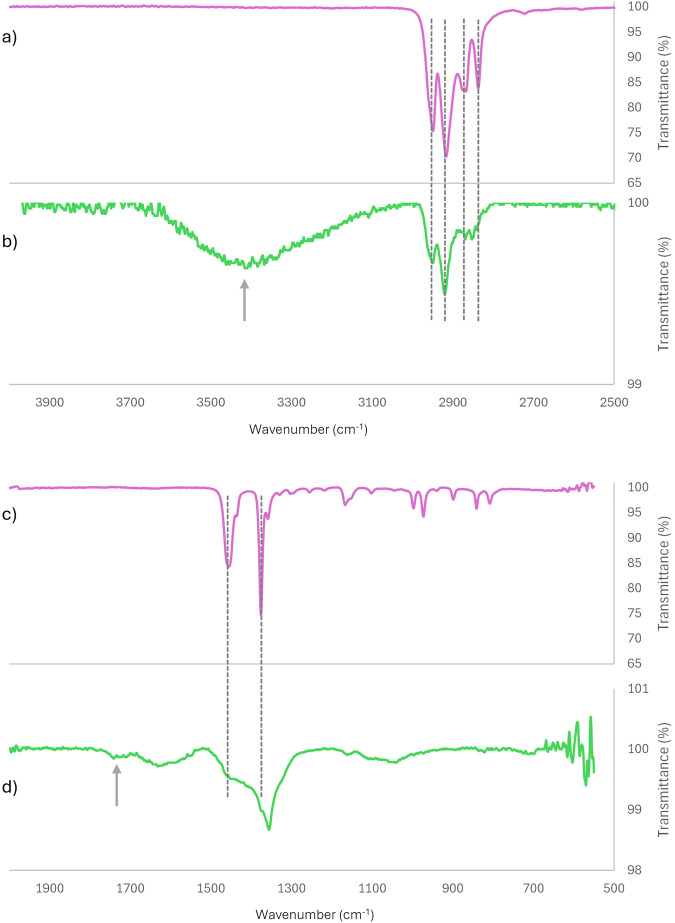
ATR-FTIR spectra of bulk material and colloidal particles after ultrasound treatment. **(a,c)** show different regions of the FTIR spectra of the bulk material, while **(b,d)** display the corresponding regions for the colloidal particles found in the control sample after ultrasound treatment of MilliQ H_2_O in the tube. The grey dotted lines indicate peak assignments for polypropylene, as referenced in sources (Hedrick and Chuang, 1998; Smith, 2021). The grey arrows highlight two regions in the spectra, suggesting that oxygen, in the form of alcohol and carboxyl groups, has been incorporated into the chemistry of the formed colloidal particles.

The control sample was also investigated with ATR-FTIR. The results indicate that the chemical composition of these particles differs from that of the bulk material of the sample vessel (see Figure 2; Supplementary Table S1). The bulk material exhibits the typical peaks for polypropylene in its FTIR spectrum (Hedrick and Chuang, 1998; Smith, 2021) and achieves a search score of 0.97 for isotactic polypropylene when compared against a polymer database consisting of 159 polymer FTIR spectra. The released particles still display characteristic peaks for CH_3_ and CH_2_ asymmetric and symmetric stretches just below 3,000 cm^-1^; however, the region below 1,500 cm^-1^ shows significant differences. Additionally, a broad peak between 3,000 and 3,500 cm^-1^ and a peak at 1740 cm^-1^ indicate the incorporation of–OH and–C=O groups into the released material’s s chemistry. A search against the same polymer database reveals that polypropylene is only ranked second, just after poly (4-methyl-1-pentene), but with low scores of around 0.40. Thus, the material detected in the solution consists of particles smaller than 400 nm, with a different chemical composition than the bulk material and, most likely, a non-uniform appearance.

**FIGURE 3:**
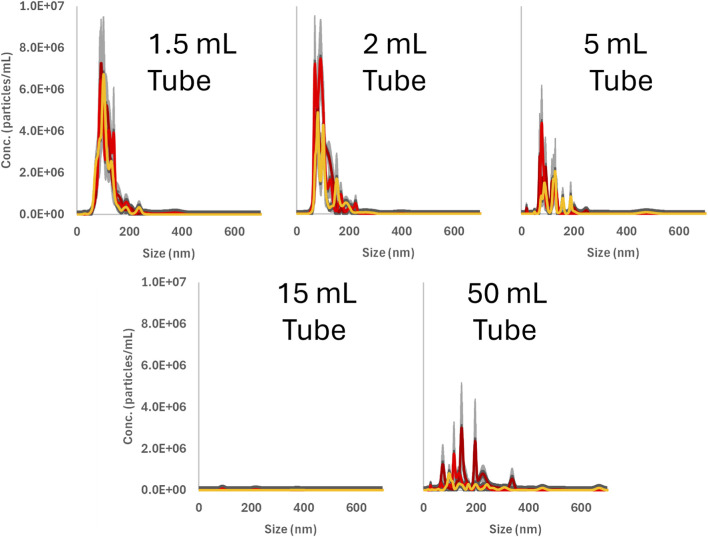
Detected nanoparticles after ultrasound treatment measured using nanoparticle tracking analysis (NTA). The dark red, red and orange lines represent the mean value from 5 individual recorded videos, camera level 14, for each of 3 sample replicates. The light grey is the error bars for each sample.

To further investigate the prevalence of nanoparticle release from plastic sample vessels, the experiment was expanded using a different ultrasound bath and sample tubes of varying sizes. The results demonstrate that the release of nanoplastics is a general phenomenon, see Figure 3. Additionally, the data indicate that particle concentrations are higher in smaller (1.5, 2.0 and 5 mL) sample vessels compared to larger (15 and 50 mL) ones, possibly due to the larger surface area-to-volume ratio. However, this finding, which warrants further investigation, is beyond the scope of this perspective.

## Discussion

The interaction between samples, for example, proteins, and the surface of the sample vessel has been identified as problematic, as highlighted in a review by Bowen and Remaley (2014). Before the advent of plastics, the scientific community predominantly relied on glass sample vessels. The composition, quality, and manufacturing techniques of glass vessels varied, meaning that even before the introduction of plastics, the experimental conditions experienced by samples differed between laboratories worldwide. Löwbeer and Diedrich (2011) addressed differences between glass and plastic tubes regarding the detection of anti-Fy^a^ in a letter to the editor in 2011. Similarly, Rolovic and Baldini (1967) investigated the impact of plastic versus glass containers on human platelet viability as early as 1967. These references, among others (Black and Kay, 1986; Feng and Wan, 1988; Nakanishi et al., 2001; Tanney et al., 2019), illustrate that concerns about the effects of sample vessels on experimental outcomes are not new.

Whether the introduction of plastics has increased or decreased this variability remains unclear. However, it is well-documented that, in *in vitro* experiments, using model polystyrene nanoparticles, engineered into spheres with uniform and narrow size distribution, can influence processes such as protein fibrillation (Cabaleiro-Lago et al., 2010a; Cabaleiro-Lago et al., 2010b; Cabaleiro-Lago et al., 2008; Cabaleiro-Lago et al., 2012; Linse et al., 2007) and the blood coagulation system (Sanfins et al., 2014). The size and surface chemistry of these nanoparticles have been shown to significantly affect experimental results.

In contrast, the particles detected in the ultrasound-treated MilliQ-H_2_O exhibit a pronounced heterogeneity in size, as demonstrated by the NTA measurements. These particles, which detach from the test tubes, are likely more similar to those formed during the breakdown of plastics, as shown, e.g., for PLA(Kelpsiene et al., 2023) and polystyrene (Ekvall et al., 2019), where the resulting fragments are typically characterized by irregular morphologies and broad size distributions, in contrast to engineered, monodisperse spherical particles.

Moreover, it should be emphasized that the particles released from the tube material do not possess the same chemical composition as the pristine bulk polymer, as illustrated in Figure 2. Our previous studies consistently demonstrate that nanoscale particles generated during macroplastic degradation differ chemically from the parent material. ATR-FTIR analyses commonly reveal the incorporation of alcohol and carboxyl functional groups into the polymer structure during the degradation process. Consequently, the released nanoparticles display surface chemistries and curvatures that diverge from those of the intact tube material, which implies that they may interact with samples in ways that differ from the unaltered tube surfaces.

In addition, we report here the effects of ultrasound treatment on commonly used polypropylene sample tubes. However, the scientific community employs tubes made from a diverse array of polymers, some of which also include commercial surface modifications intended to reduce, for example, nonspecific adsorption of proteins or nucleic acids. Whether such tubes similarly release nanoscale particles under ultrasound exposure—or under other conditions involving mechanical or thermal energy input, such as tip sonication, agitation, or heating—and whether surface modifications influence the size, morphology, or chemical characteristics of the released particles lie beyond the scope of this brief report.

To conclude, should we be concerned about plastic nanoparticles from our sample vessels?

An estimate of the additional surface area contributed by particles formed during ultrasonication in our initial experiment (see Figure 1) suggests an approximately 10% increase relative to the vessel’s original surface area for a 5 mL sample vessel. For a 1.5 mL sample vessel, the increase is approximately 20%. These values may be underestimated, as NTA does not detect polymer particles smaller than ∼50 nm (Filipe et al., 2010; Malloy and Carr, 2006), which can be seen in Figures 1, 3.

While these increases are not substantial, the nanoparticles exhibit distinct structural and chemical properties compared to the sample vessel surface. It is also important to emphasize that the main data reported in this perspective originate from an experimental setup designed to study the release of small molecules and nanoparticles from EPDM granules, in which extended ultrasound treatments (totaling 30 h) were applied. Supplementary Figure S2 presents NTA data comparing a sample subjected to 30 min of ultrasound treatment with a sample left on the laboratory bench for 30 min. The results show that the number of detected particles more than doubles and that the size distribution becomes more complex; however, the particle counts remain well below those observed after 30 h of ultrasound treatment.

In this article, we have highlighted that ultrasound can promote the release of nanoplastics from the sample tube walls. However, other techniques in the lab could potentially have similar effects, for example, vortex or freeze-thaw cycles. The phenomenon of unwanted particles from our laboratory material and tools is not a new observation for us. We have observed in other studies that sonication using a tip resulted in the release of metal particles from the tip into the solution. In that case, we could remove most of the metal particles by centrifugation, however, at that time, we did not know about the plastic nanoparticles from the sample tube. Given that differences in size, structure, and chemistry of nanoparticles have repeatedly been shown to significantly affect their interactions with samples, we believe the detected particles highlighted in this perspective warrant further attention from the research community.

## Data Availability

The raw data supporting the conclusions of this article will be made available by the authors, without undue reservation.

## References

[B1] Bhan A. Delgass W. N. (2022). Best practices in catalysis: a perspective. J. Catal. 405, 419–429. 10.1016/j.jcat.2021.12.014

[B2] Black D. Kay J. (1986). Influence of tube type on the antiglobulin-test. Med. Lab. Sci. 43, 169–173. 3736368

[B3] Bowen R. A. R. Remaley A. T. (2014). Interferences from blood collection tube components on clinical chemistry assays. Biochem. Medica 24, 31–44. 10.11613/BM.2014.006 24627713 PMC3936985

[B4] Cabaleiro-Lago C. Quinlan-Pluck F. Lynch I. Lindman S. Minogue A. M. Thulin E. (2008). Inhibition of amyloid β protein fibrillation by polymeric nanoparticles. J. Of Am. Chem. Soc. 130, 15437–15443. 10.1021/ja8041806 18954050

[B5] Cabaleiro-Lago C. Lynch I. Dawson K. A. Linse S. (2010a). Inhibition of IAPP and IAPP (20-29) fibrillation by polymeric nanoparticles. Langmuir 26, 3453–3461. 10.1021/la902980d 20017535

[B6] Cabaleiro-Lago C. Quinlan-Pluck F. Lynch I. Dawson K. A. Linse S. (2010b). Dual effect of amino modified polystyrene nanoparticles on amyloid β protein fibrillation. ACS Chem. Neurosci. 1, 279–287. 10.1021/cn900027u 22778827 PMC3368671

[B7] Cabaleiro-Lago C. Szczepankiewicz O. Linse S. (2012). The effect of nanoparticles on amyloid aggregation depends on the protein stability and intrinsic aggregation rate. LANGMUIR 28, 1852–1857. 10.1021/la203078w 22168533 PMC3265146

[B12] Chandra P. Enespa, Singh R. Arora P. K. (2020). Microbial lipases and their industrial applications: a comprehensive review. Microb. Cell Factories 19, 169. 10.1186/s12934-020-01428-8 32847584 PMC7449042

[B8] Chen G. Feng Q. Wang J. (2020). Mini-review of microplastics in the atmosphere and their risks to humans. Sci. Total Environ. 703, 135504. 10.1016/j.scitotenv.2019.135504 31753503

[B9] Du J. Zhou Q. Li H. Xu S. W C. Fu L. (2021). Environmental distribution, transport and ecotoxicity of microplastics: a review. J. Appl. Toxicol. 41, 52–64. 10.1002/jat.4034 32671862

[B10] Dusselier M. Sels B. F. (2014). “Selective catalysis for cellulose conversion to lactic acid and other α-Hydroxy acids,” in Selective catalysis for renewable feedstocks and chemicals. Editor NICHOLAS K. M. (Cham: Springer International Publishing). 10.1007/128_2014_540 24824728

[B11] Ekvall M. T. Lundqvist M. Kelpsiene E. Šileikis E. Gunnarsson S. B. Cedervall T. (2019). Nanoplastics formed during the mechanical breakdown of daily-use polystyrene products. Nanoscale Adv. 1, 1055–1061. 10.1039/c8na00210j 36133186 PMC9473236

[B13] Feng C.-S. Wan C.-P. (1988). The plastic tube compared with the glass tube in blood bank tests. Pathology 20, 309–310. 10.3109/00313028809059518 3205604

[B14] Filipe V. Hawe A. Jiskoot W. (2010). Critical evaluation of nanoparticle tracking analysis (NTA) by NanoSight for the measurement of nanoparticles and protein aggregates. Pharm. Res. 27, 796–810. 10.1007/s11095-010-0073-2 20204471 PMC2852530

[B15] Ge J. Li H. Liu P. Zhang Z. Ouyang Z. Guo X. (2021). Review of the toxic effect of microplastics on terrestrial and aquatic plants. Sci. Total Environ. 791, 148333. 10.1016/j.scitotenv.2021.148333 34412379

[B16] Gigault J. Halle A. T. Baudrimont M. Pascal P. Y. Gauffre F. Phi T. L. (2018). Current opinion: what is a nanoplastic? Environ. Pollut. 235, 1030–1034. 10.1016/j.envpol.2018.01.024 29370948

[B17] Gigault J. El Hadri H. Nguyen B. Grassl B. Rowenczyk L. Tufenkji N. (2021). Nanoplastics are neither microplastics nor engineered nanoparticles. Nat. Nanotechnol. 16, 501–507. 10.1038/s41565-021-00886-4 33927364

[B18] Hedrick S. A. Chuang S. S. C. (1998). Temperature programmed decomposition of polypropylene: *in situ* FTIR coupled with mass spectroscopy study. Thermochim. Acta 315, 159–168. 10.1016/s0040-6031(98)00283-4

[B19] Hernandez L. M. Xu E. G. Larsson H. C. E. Tahara R. Maisuria V. B. Tufenkji N. (2019). Plastic teabags release billions of microparticles and nanoparticles into tea. Environ. Sci. and Technol. 53, 12300–12310. 10.1021/acs.est.9b02540 31552738

[B20] Jenner L. C. Rotchell J. M. Bennett R. T. Cowen M. tentzeris V. Sadofsky L. R. (2022). Detection of microplastics in human lung tissue using μFTIR spectroscopy. Sci. Total Environ. 831, 154907. 10.1016/j.scitotenv.2022.154907 35364151

[B21] Kelpsiene E. Rydberg M. Ekvall M. T. Lundqvist M. Cedervall T. (2023). Prolonged survival time of Daphnia magna exposed to polylactic acid breakdown nanoplastics. PLOS ONE 18, e0290748. 10.1371/journal.pone.0290748 37669271 PMC10479899

[B22] Leonard S. V. L. Liddle C. R. Atherall C. A. Chapman E. Watkins M. Calaminus S. D. J. (2024). Microplastics in human blood: polymer types, concentrations and characterisation using μFTIR. Environ. Int. 188, 108751. 10.1016/j.envint.2024.108751 38761430

[B23] Li W. Wang H. Jiang X. Zhu J. Liu Z. Guo X. (2018). A short review of recent advances in CO2 hydrogenation to hydrocarbons over heterogeneous catalysts. RSC Adv. 8, 7651–7669. 10.1039/c7ra13546g 35539148 PMC9078493

[B24] Linse S. Cabaleiro-Lago C. Xue W. F. Lynch I. Lindman S. Thulin E. (2007). Nucleation of protein fibrillation by nanoparticles. Proc. Of Natl. Acad. Of Sci. Of U. S. A. 104, 8691–8696. 10.1073/pnas.0701250104 17485668 PMC1866183

[B25] Löwbeer C. Diedrich B. (2011). A comparison between different glass and plastic tubes regarding the detection of Anti-Fya. Transfus. Med. 21, 134–136. 10.1111/j.1365-3148.2010.01046.x 21039981

[B26] Lynch I. Cedervall T. Lundqvist M. Cabaleiro-Lago C. Linse S. Dawson K. A. (2007). The nanoparticle - protein complex as a biological entity; a complex fluids and surface science challenge for the 21st century. Adv. Colloid Interface Sci. 134-35, 167–174. 10.1016/j.cis.2007.04.021 17574200

[B27] Malloy A. Carr B. (2006). NanoParticle tracking analysis – the halo™ system. Part. and Part. Syst. Charact. 23, 197–204. 10.1002/ppsc.200601031

[B28] Nakanishi K. Sakiyama T. Imamura K. (2001). On the adsorption of proteins on solid surfaces, a common but very complicated phenomenon. J. Biosci. Bioeng. 91, 233–244. 10.1263/jbb.91.233 16232982

[B29] Ragusa A. Svelato A. Santacroce C. Catalano P. Notarstefano V. Carnevali O. (2021). Plasticenta: first evidence of microplastics in human placenta. Environ. Int. 146, 106274. 10.1016/j.envint.2020.106274 33395930

[B30] Rolovic Z. Baldini M. (1967). Comparative studies of the viability of human platelets stored at 4 C in plastic and glass containers. Transfusion 7, 204–211. 10.1111/j.1537-2995.1967.tb05511.x 6025695

[B31] Sanfins E. Augustsson C. Dahlbäck B. Linse S. Cedervall T. (2014). Size-dependent effects of nanoparticles on enzymes in the blood coagulation Cascade. NANO Lett. 14, 4736–4744. 10.1021/nl501863u 25025946

[B32] Smith B. C. (2021). The infrared spectra of polymers III: hydrocarbon polymers. Spectroscopy 36, 22–25. 10.56530/spectroscopy.mh7872q7

[B33] Tanney K. Mahaveer A. Dockery K. Booth N. Chadwick C. A. Chaloner C. M. (2019). Non-reassuring results in agreement trial comparing glass and plastic capillary tubes for neonatal blood gas sampling. Acta Paediatr. 108, 1055–1060. 10.1111/apa.14653 30456830

[B34] Ten Hietbrink S. Materić D. Holzinger R. Groeskamp S. Niemann H. (2025). Nanoplastic concentrations across the north Atlantic. Nature 643, 412–416. 10.1038/s41586-025-09218-1 40634739 PMC12240857

[B35] Vega-Herrera A. Garcia-Torné M. Borrell-Diaz X. Abad E. Llorca M. Villanueva C. M. (2023). Exposure to micro(nano)plastics polymers in water stored in single-use plastic bottles. Chemosphere 343, 140106. 10.1016/j.chemosphere.2023.140106 37689148

[B36] Vogt C. Weckhuysen B. M. (2022). The concept of active site in heterogeneous catalysis. Nat. Rev. Chem. 6, 89–111. 10.1038/s41570-021-00340-y 37117296

[B37] Yang H. Niu S. Guo M. Xue Y. (2025). A critical review of the ecotoxic effects of microplastics on aquatic, soil and atmospheric ecosystems and current research challenges. Environ. Res. 274, 121361. 10.1016/j.envres.2025.121361 40068785

[B38] Zhu W. Zhang R. Qu F. Asiri A. M. Sun X. (2017). Design and application of foams for electrocatalysis. ChemCatChem 9, 1721–1743. 10.1002/cctc.201601607

