# Supplementary material for: On the background of plastics nanoparticles in our research
Source: Front Toxicol. 2026 Apr 23;8:1653106. doi: 10.3389/ftox.2026.1653106 (PMC13148795; doi:10.3389/ftox.2026.1653106)
Supplement: Supplementary file 1 [file Supplementaryfile1.pdf]

Supplementary material:

On the background of plastics nanoparticles in our research.

Martin Lundqvist[1]*, Jing Hua[1], Mikael T. Ekvall[1], and Tommy Cedervall[1]

[1]Biochemistry and Structural Biology, Lund University, Lund, Sweden

*Corresponding author

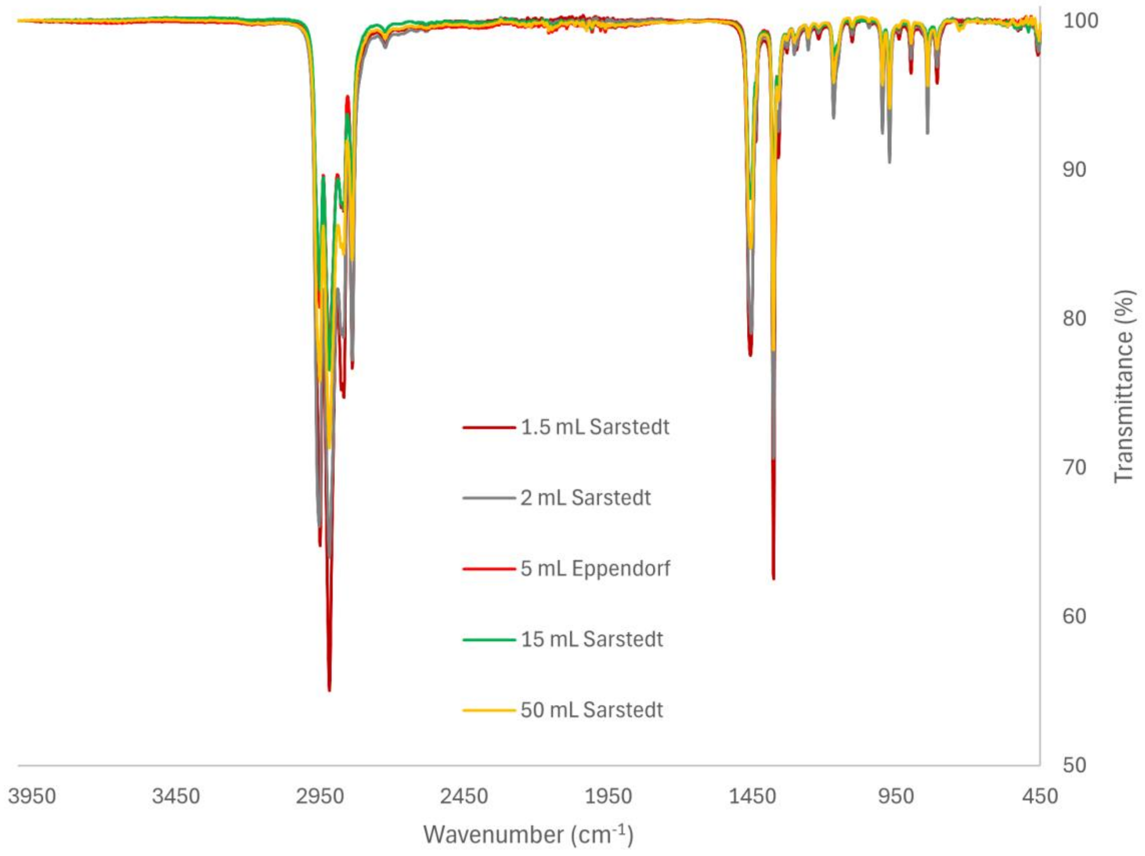

Figure S1. FTIR spectra of the bulk material of 5 of the 6 tested tubes. A piece of each tube was cut and placed with the inside toward the crystal at the ATR unit. The FTIR setup was 4 scans, with 4 cm^{-1} resolution between 4000-450 cm^{-1}. The spectra have been baseline corrected. All tubes were made of polypropylene, see Table S1.

Table S1. FTIR spectra Library[1] search

**Sample Name**	Search Score	Search Best Hit Description	Search Library[1]
Bulk 1.5 mL Sarstedt	0.95609	POLYPROPYLENE, ISOTACTIC	polyatr
Bulk 15 mL Sarstedt	0.964017	POLYPROPYLENE, ISOTACTIC	polyatr
Bulk 2 mL Sarstedt	0.970188	POLYPROPYLENE, ISOTACTIC	polyatr
Bulk 5 mL Eppendorf	0.973351	POLYPROPYLENE, ISOTACTIC	polyatr
Bulk 50 mL Sarstedt	0.972135	POLYPROPYLENE, ISOTACTIC	polyatr
		Search Reference Spectrum Description	
Colloidal particles formed after ultrasound treatment	0.404913	POLY(4-METHYL-1-PENTENE)MELT INDEX 70	polyatr
Colloidal particles formed after ultrasound treatment	0.393731	POLYPROPYLENE, ISOTACTIC	polyatr

[1] A library containing 159 reference IR spectra of polymers that was bought together with the instrument from PerkinElmer.

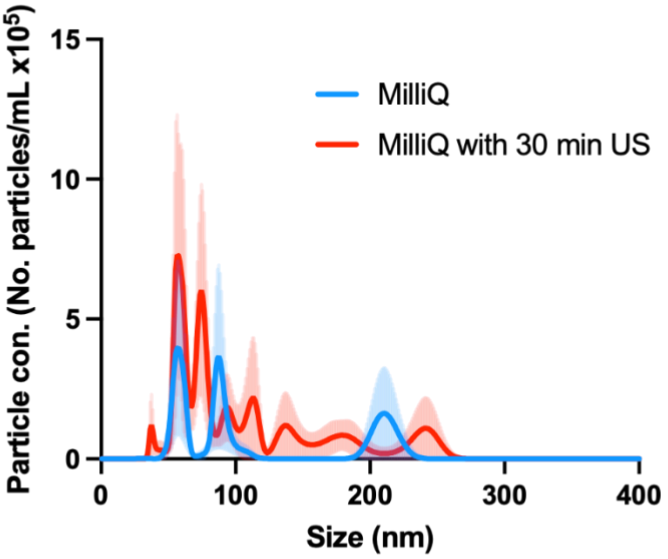

Figure S2. Nanoparticle tracking analysis (NTA) data for a 5 mL test tube containing Milli-Q water subjected to ultrasound treatment. Data are shown for a tube filled with Milli-Q water and left to stand for 30 minutes before measurement (blue), and after 30 minutes of ultrasound treatment (red). The blue and red lines represent the mean particle size distributions from five individual recordings, and the shaded areas indicate the corresponding standard deviations.
